# Supplementary material for: Effect of Surface Coating of Gold Nanoparticles on Cytotoxicity and Cell Cycle Progression
Source: Nanomaterials (Basel). 2018 Dec 17;8(12):1063. doi: 10.3390/nano8121063 (PMC6316730; doi:10.3390/nano8121063)
Supplement: Supplementary file 1 [file nanomaterials-08-01063-s001.pdf]

Effect of Surface Coating of Gold Nanoparticles on Cytotoxicity and Cell Cycle Progression

Qian Li, Chun Huang, Liwei Liu, Rui Hu * and Junle Qu

Key Laboratory of Optoelectronic Devices and Systems of Ministry of Education and Guangdong Province, College of Optoelectronic Engineering, Shenzhen University, Shenzhen 518060, China; liqian123@szu.edu.cn (Q.L.); huangchun1190@163.com (C.H.); liulw@szu.edu.cn (L.L.); jlqu@szu.edu.cn (J.Q.)

* Correspondence:rhu@szu.edu.cn; Tel.: +86-0755-2673-3319; Fax: +86-0755-2653-6237

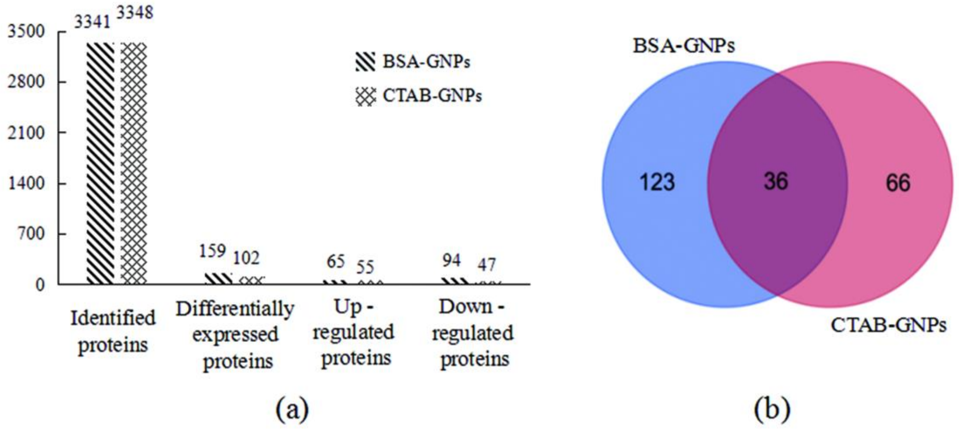

Figure S1. Identified and differentially expressed proteins in GNPs treated raw 264.7 cells from. iTRAQ proteomics. (**a**) Identified proteins and differentially accumulated proteins from iTRAQ proteomics. Differentially accumulated proteins analysis based on the fold-change >1.5 or <0.667 (P < 0.05); down-regulation proteins with fold-change >1.5 (P < 0.05); up-regulation proteins with fold-change <0.667 (P < 0.05). (**b**) A Venn diagram showing the overlap of differentially expressed proteins between BSA-GNPs and CTAB-GNPs.

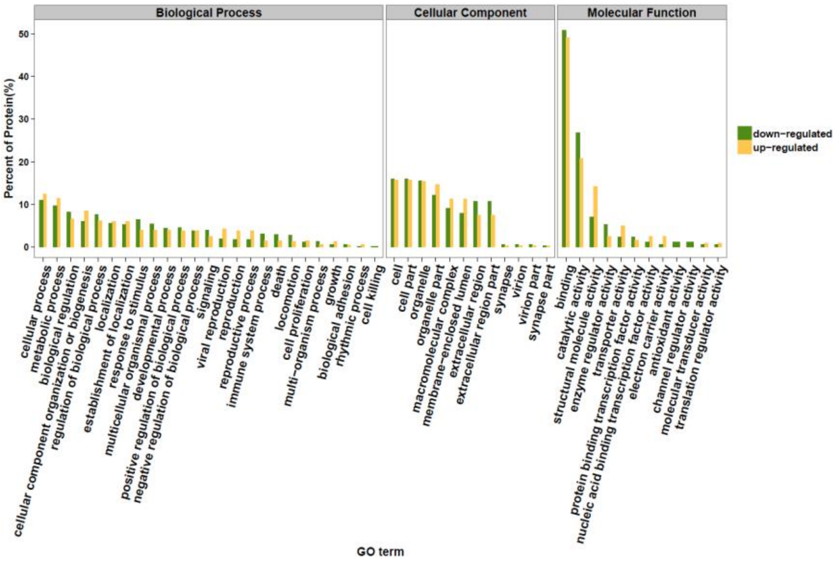

(a)

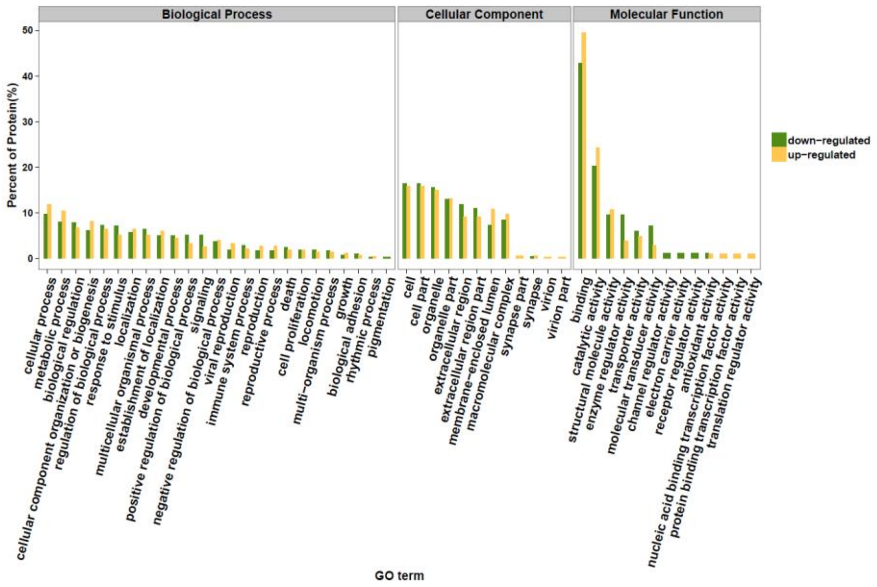

(b)

Figure S2. Gene ontology (GO) classification of differential expressed proteins in (**a**) BSA-GNPs and (**b**) CTAB-GNPs. GO classifications of differentially expressed proteins were divided into three classes including biological processes, cellular components, and molecular functions.

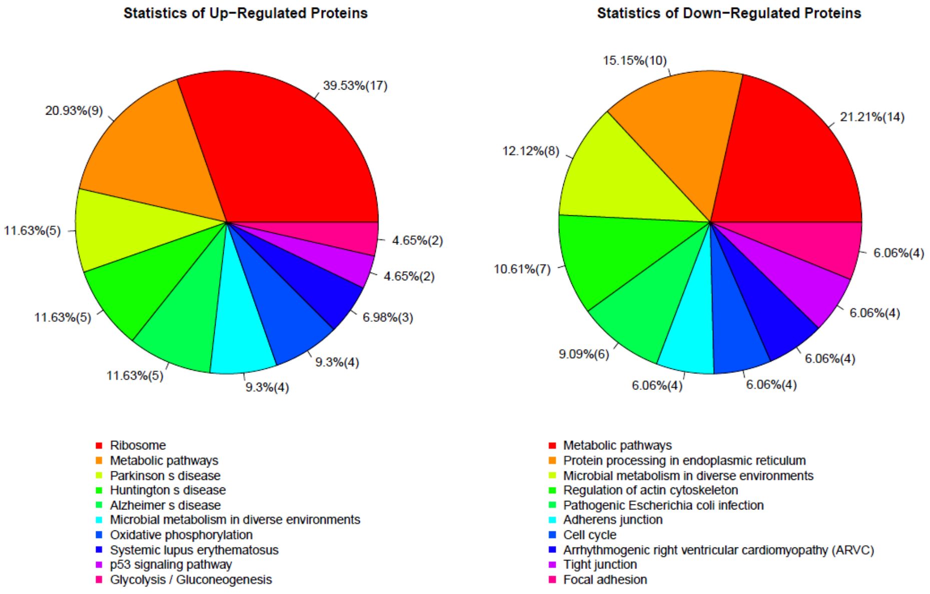

(a)

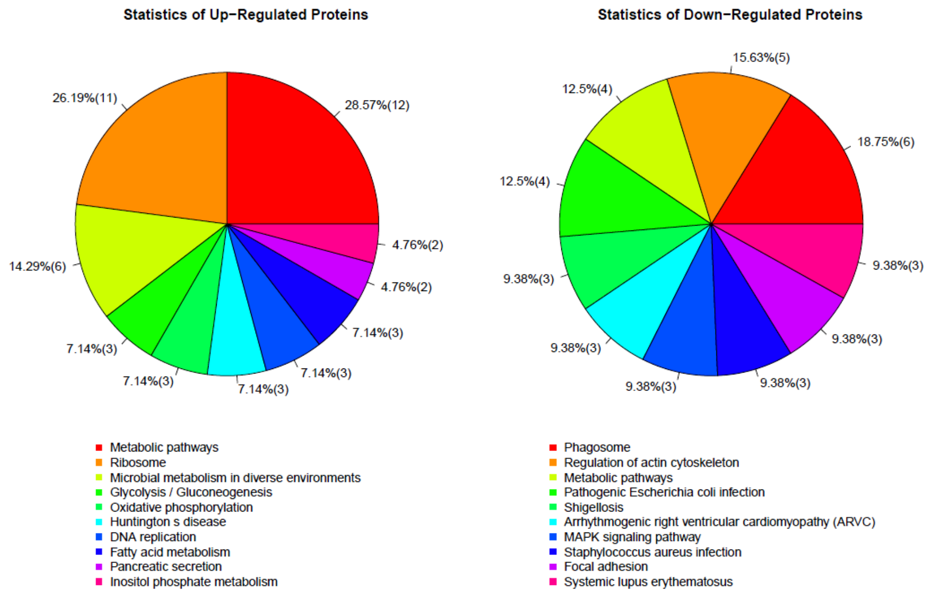

(b)

Figure S3. Top 10 changed pathways based on proteome analysis in (**a**) BSA-GNPs and (**b**) CTAB-GNPs. Down-regulation of cell cycle-related proteins was observed following BSA-GNP treatment. Down-regulation of actin cytoskeleton-related proteins, which are closely related to the cell cycle, wereobserved following CTAB-GNP treatment.
